# Influences of Different Air-Inhibition Coatings on Monomer Release, Microhardness, and Color Stability of Two Composite Materials

**DOI:** 10.1155/2019/4240264

**Published:** 2019-05-09

**Authors:** Luca Marigo, Giuseppina Nocca, Giulia Fiorenzano, Cinzia Callà, Raffaella Castagnola, Massimo Cordaro, Gaetano Paolone, Salvatore Sauro

**Affiliations:** ^1^UOC Odontoiatria Generale e Ortodonzia, Dip. Scienze dell'Invecchiamento, Neurologiche, Ortopediche e della Testa Collo. Fondazione Policlinico Universitario A. Gemelli, IRCCS, Roma 00168, Italy; ^2^Istituto di Clinica Odontoiatrica, Università Cattolica del Sacro Cuore, Roma 00168, Italy; ^3^Istituto di Biochimica e Biochimica Clinica, Università Cattolica del Sacro Cuore, Roma 00168, Italy; ^4^Fondazione Policlinico Universitario A. Gemelli, IRCCS, Roma 00168, Italy; ^5^UOC Chimica, Biochimica e Biologia Molecolare, Dip. Scienze di Laboratorio e Infettivologiche, Fondazione Policlinico Universitario A. Gemelli, IRCCS, Roma 00168, Italy; ^6^Dental school, San Raffaele University, Milan 20132, Italy; ^7^Departamento de Odontología, Facultad de Ciencias de la Salud, Universidad CEU-Cardenal Herrera C/Del Pozo s/n, Alfara del Patriarca, Valencia 46115, Spain; ^8^Department of Therapeutic Dentistry, Sechenov University Russia, Moscow 119435, Russia

## Abstract

The aim of this study was to evaluate the effect of light-curing protocols on two modern resin composites using different air-inhibition coating strategies. This was accomplished by assessing the amount of monomer elution, surface microhardness, and composite discoloration in different storage conditions. A total of 120 specimens were prepared using Filtek Supreme XTE (3M ESPE, Seefeld, Germany) and CeramX Universal (Dentsply DeTrey, Konstanz, Germany). Specimens were light-cured in air as per manufacturer's instructions or in the absence of oxygen. This latter condition was achieved using three different approaches: (i) transparent polyester strip; (ii) glycerin; (iii) argon gas. Specimens were assessed for release of monomers, Vickers hardness, and discoloration after storage in different solutions. The results were analyzed with ANOVA one-way test followed by Student-Newman-Keuls test. Moreover, multiple comparisons of means were performed using the Student t-test (p<0.05). The amount of monomers released from the tested specimens was very low in all conditions. The presence of oxygen induced some decrease in microhardness. The highest discoloration values, for both materials, were obtained after ageing in red wine. In case finish and polish procedures are awkward to achieve in posteriors composite restoration, light-curing in the absence of oxygen should be considered, especially when performing composite restoration in esthetic areas.

## 1. Introduction

Composite resins have been radically improved in the last years in terms of physical properties and aesthetic characteristics [1, 2]. Nowadays, nanohybrid and nanofilled composites are considered universal resin-based restorative materials suitable for the restoration of anterior and posterior teeth due to their excellent aesthetic properties [3].

Nevertheless, their polymerization reaction can be inhibited during light-curing procedures due to the presence of oxygen in the atmosphere. This latter acts as scavenger, which tends to convert highly reactive radicals into relatively stable hydroperoxides. The presence of these latter components can alter the quality of the polymerization of the outer layer of resin composites [4]. This results in a sticky superficial layer on the outer surface of resin composites, which is rich in unreacted monomers; it is known as oxygen inhibited layer (OIL) [5–7].

Due to the importance of the polymerization reaction for the hardness and monomer elution, as well as for aesthetic discoloration of composite resins, it is mandatory, especially in clinical practice, to optimize the polymerization reaction conditions [8, 9]. During light-curing processes, an air-inhibition coating can be used to reduce the OIL [3, 10]. Transparent polyester strip (mylar strip) as well as the use of a layer of glycerin accomplishes such a purpose. Indeed, Mylar strip and glycerin can act as physical barriers once placed on the surface of the resin before the light-curing procedures. Conversely, in the presence of an argon-rich atmosphere, the formation of free radicals is drastically reduced during the polymerization reaction. This happens because argon atoms bind and inhibit such radicals and the degree of conversion of monomers to polymers is enhanced [5].

A suitable method to evaluate the effect of oxygen on the polymerization of resin composites is through high-performance liquid chromatography (HPLC), which is widely used to determine the amount of monomers eluted from the resin matrix during water storage [11]. One more method usually employed to assess the efficiency of light-curing procedures on dental composites is based on the evaluation of Vickers microhardness [12]. Moreover, color stability of resin composites is essential to achieve and maintain acceptable aesthetic goals in direct restorative dentistry. Indeed, by using a spectrophotometer, it may be possible to “translate” the color in coordinates and calculate the staining effects induced by different staining solutions, (e.g., coffee, red wine, tea) [13–15].

The aim of this study was to evaluate the effect of light-curing procedures on two modern resin composites using different air-inhibition coating strategies. This was accomplished by assessing the amount of monomer elution, surface microhardness, and composite discoloration in different storage conditions.

The hypotheses of this study were that the polymerization performed in the absence of oxygen would (a) increase the chemicophysical properties of the tested composite such as microhardness and monomer elution and (b) increase the stability of the composite discoloration when stored in different staining solution (red wine, coffee, and distilled water).

## 2. Materials and Methods

### 2.1. Experimental Design

Sixty specimens for each material (total number: 120) were prepared using a stainless-steel mold to obtain disc-shape specimens (diameter: 6.5 mm; thickness 2 mm). A nanofilled (Filtek Supreme XTE, 3 M ESPE, Seefeld, Germany) (XTE) and a nanohybrid composite (CeramX Universal, Dentsply DeTrey, Konstanz, Germany) (CX) (Table 1) with a standardized initial shade A2 (Vita Shade guide, Vita ZahnFabrik, Bad Säckingen, Germany) were used in this study. Light-curing procedures were performed using a light-emitting diode (LED) polymerization system (BlancOne® IDS, with 2200 mW/cm^2^ light intensity) according to the manufacturer's instructions (20 sec), with the light at 1 mm of distance and perpendicular to the surface of the specimens.

The specimens were divided into four main groups based on different light-curing conditions (15sp/group).

*Group A*. Specimens polymerized under a 0.05 mm-thick Mylar strip (Westpoint, Firenze, Italy), which was applied on the surface of the composite prior to light-curing procedures.

*Group B*. Specimens polymerized using a thin layer of glycerin (Shiny G Air block in tips 0,3 g, Micerium, Avegno, Italia) applied on the surface of the composite prior to light-curing procedures. After polymerization, glycerin was removed with ethanol.

**Figure 1:**
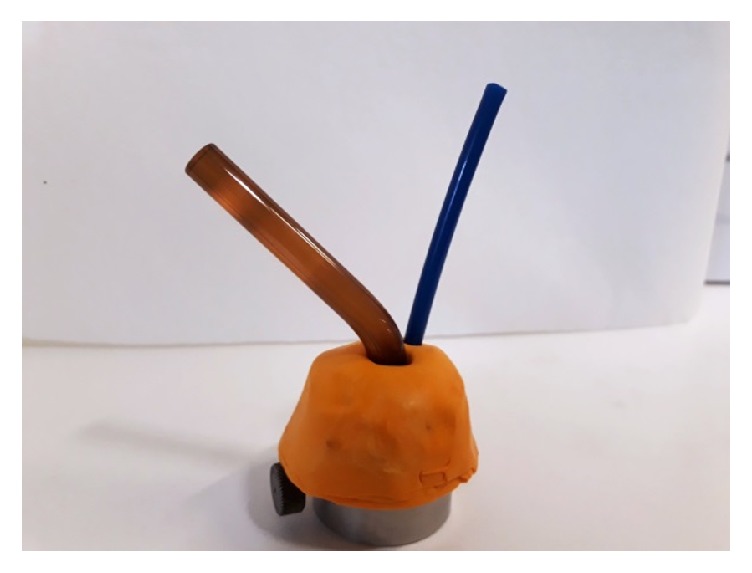
The argon chamber device.

*Group C*. Specimens polymerized in the presence of argon-rich atmosphere using a customized chamber created with high viscosity silicone; this allowed the argon gas to diffuse and replace the oxygen during the light-curing procedures (Figure 1).

*Group D*. Specimens polymerized without any barrier between the surface of the resin composite and the light-curing tip.

### 2.2. High-Performance Liquid Chromatography: Evaluation of Eluates

Three specimens for each group (twelve for each material) were used to evaluate the elution of monomers after different light-curing procedures. The HPLC was employed to determine the amount of monomers leached out from the specimens after storage, immersion in ethanol (2.8 mL), and incubation for 24 h at 37°C [16, 17]. This incubation time was selected on the basis of a pilot study that have confirmed that the most monomers are eluted in the first 24 hours (data not shown). The supernatant was then centrifuged and filtered (0.45 *μ*m syringe filter; Whatman, Maidstone, Kent, UK). This was subsequently analyzed using a JASCO (Easton, MD, USA) HPLC system (2 PU-980 pumps, UV-970 UV/VIS detector, and AS-1555 autosampler). The analysis was performed at a wavelength of 214 nm with a C-18 (5 *μ*m) Supelco reversed phase column (250 × 4.6 mm) using an elution gradient of water (A) and acetonitrile (B) starting from 40% to 20% of A (30min), 0.7 mL/min flow, 50 *μ*L injected volume.

The concentration of triethylene glycol dimethacrylate (TEGDMA), diurethane dimethacrylate (DUDMA), and bisphenol-A-glycidyl-methacrylate (bis-GMA) released into the ethanol was quantified before and after each analysis and compared to the values of a calibration line, previously created using standard solutions (Sigma Aldrich, Milan, Italy).

### 2.3. Vickers Microhardness Evaluation

Three specimens for each group (twelve for each material) were analyzed to evaluate the Vickers microhardness (Microhardness Tester MHT4, Zeiss, Jana, Germany), with 100 g load (0.981 N) and 10 s duel-time (slope: 10 gf/s). Three indentations were recorded for each specimen at different areas of the outer surface. The mean value was then calculated and converted into a Vickers hardness number (VHN) as described in previous studies [18]. VHN values were expressed as N/mm^2^ (MPa).

### 2.4. Spectrophotometric Analysis: Discoloration Assessment

**Figure 2:**
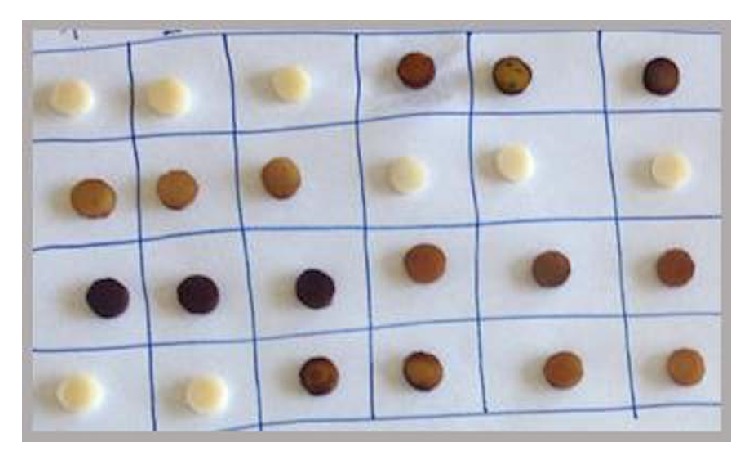
The discoloration endpoints of different specimens.

Nine specimens for each polymerization protocol (36 for each composite material) were used for the discoloration assessment through spectrophotometric analysis using VITA EasyShade® Compact (Vita ZahnFabrik, Bad Säckingen, Germany). The specimens were subjected to colorimetric evaluation after 24 h of incubation in distilled water (t_0_) and after immersion in one of the following solutions: distilled water (control group), red wine (Sangiovese di Romagna DOP, Bologna, Italy), and coffee (Nescafé® Gran Aroma coffee soluble, 3 g in 100 mL of hot distilled water) (Figure 2).

The specimens were then stored for 28 d at 37°C in the dark, with the staining solutions replaced every week to avoid excessive bacterial proliferation. After 28 d (t_1_), specimens were rinsed with distilled water for 2 min and dried with absorbent paper followed by 12 h in a desiccator chamber. The evaluation of the specimens was performed on white background (WB) and black background (BB) to simulate the conditions of an incisal (BB) and interproximal or occlusal restorations (WB) [19].

For each specimen, 3 measurements (single mode repeated 3 times) were performed in WB and 3 in BB. All measurements were performed by the same operator, and the instrument (shade guide) was calibrated every 10 measurements.

Subsequently, the spectrophotometer measurements were repeated and the color differences between the measurement data at t_0_ and t_1_ were calculated. According to CIE L*∗*a*∗*b*∗* color system, the color variation can be obtained using a system of coordinates of the CIE L*∗*a*∗*b*∗* scale: L (lightness, 0–100), a (−a*∗* = green, +a*∗* = red), and b (−b*∗* = blue, +b*∗* = yellow). So, the color variation ΔE of each specimen was calculated using the following equation: ΔE = [(L_1_*∗* - L_0_*∗*)^2^+ (a_1_*∗* - a_0_*∗*)^2^+ (b_1_*∗* - b_0_*∗*)^2^]^1/2^.

ΔE<1.1 is not perceptible to the human eye, while values of ΔE>3.3 correspond to visually perceptible differences considered clinically unacceptable [20].

### 2.5. Statistical Analysis

All the results were expressed in mean (M) ± standard deviation (±SD) and statistically analyzed using one-way ANOVA followed by a multiple comparison using the Student-Newman-Keuls test. When necessary, the results were also compared using the Student t-test (significance: p<0.05).

## 3. Results

**Figure 3:**
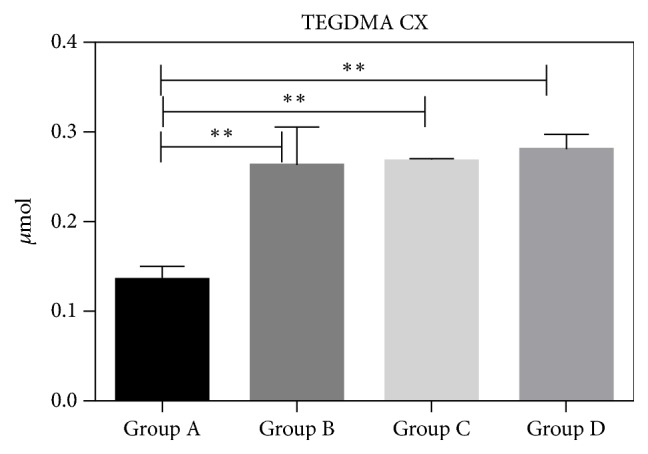
TEGDMA micromoles released by CX disks after 24 h incubation in ethanol. Three specimens for each group (twelve for each material) were used to evaluate the elution of monomers after different light-curing procedures. The concentration of TEGDMA released into the ethanol was quantified before and after each analysis and compared to the values of a calibration line. The error bars represent the standard deviation of measurements for 3 specimens in 3 separate sample runs (n = 3). Data are expressed as mean ± SD. *∗∗* p <0.01.

**Figure 4:**
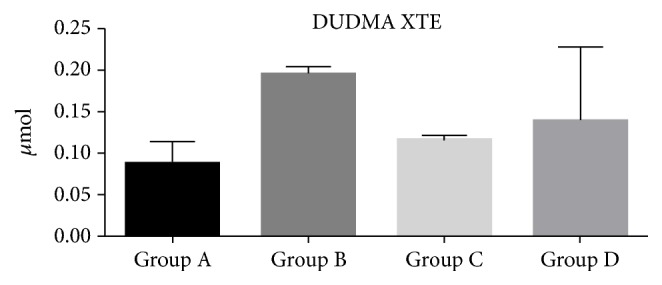
DUDMA micromoles released after 24 h of incubation in ethanol from XTE disks. Three specimens for each group (twelve for each material) were used to evaluate the elution of monomers after different light-curing procedures. The concentration of DUDMA released into the ethanol was quantified before and after each analysis and compared to the values of a calibration line. The error bars represent the standard deviation of measurements for 3 specimens in 3 separate sample runs (n = 3). Data are expressed as mean ± SD.

**Figure 5:**
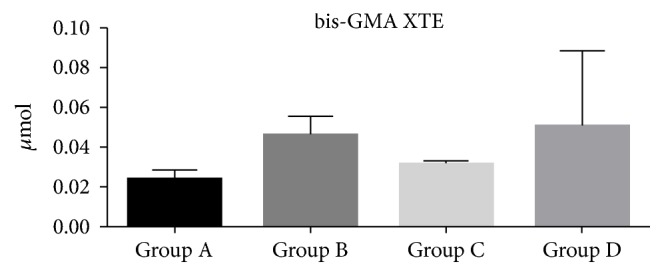
Bis-GMA micromoles released after 24 h ethanol incubation from XTE disks. Three specimens for each group (twelve for each material) were used to evaluate the elution of monomers after different light-curing procedures. The concentration of Bis-GMA released into the ethanol was quantified before and after each analysis and compared to the values of a calibration line. The error bars represent the standard deviation of measurements for 3 specimens in 3 separate sample runs (n = 3). Data are expressed as mean ± SD.

The HPLC results showed that the amount of monomers released from the composite specimens was very low in all polymerization conditions (Figures 3, 4, and 5). Overall, the presence of DUDMA and bis-GMA was mainly detected for the XTE specimens, while TEGDMA was found principally in the eluate of the CX specimens. In detail, the CX specimens light-cured with the use of the mylar matrix (Group A) showed the lowest amount of TEGDMA (p<0.01) compared to all the tested groups (Figure 3). The specimens created using XTE had no significant release of DUDMA and bis-GMA regardless the polymerization protocol employed (p> 0.05) (Figures 4 and 5).

**Figure 6:**
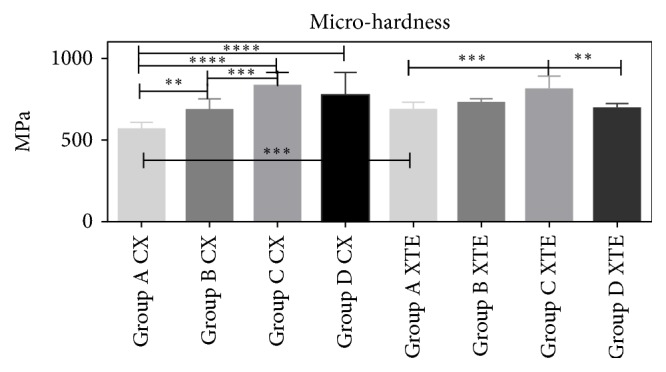
Microhardness of the specimens under different polymerization conditions. Three specimens for each group (n=3; twelve for each material) were analyzed to evaluate surface using a Vickers hardness tester. Three indentations were recorded for each specimen. The mean value was then calculated and converted into a Vickers hardness number (VHN). VHN values were expressed as N/mm2 (MPa). The error bars represent the standard deviation of measurements for 3 specimens in 3 separate determinations (n = 3). Data are expressed as mean ± SD. *∗∗* p<0.01, *∗∗∗*p<0.001, and *∗∗∗∗*p<0.0001.

The microhardness results are depicted in Figure 6. It was observed that CX light-cured using the Mylar matrix (Group A) had significantly lower hardness values (570 MPa) (p<0.01 vs Group B, p<0.0001 vs Group C, p<0.0001 vs Group D) compared to all the specimens light-cured in different polymerization conditions. Regarding XTE, the best performance was achieved in argon-rich atmosphere (812 MPa) (Group C) compared to mylar (Group A) (p<0.001) and air (Group D) (p<0.01). Significant differences were found between CX and XTE light-cured under mylar matrix (Groups A) (p<0.001).

The mean ΔE values and the statistical analysis after 28 d of immersion in the different staining solutions are shown in Table 2. When analyzing the color change of each material, CX presented the lowest degree of staining compared to XTE, regardless of the staining condition.

Red wine and coffee induced a significant increase in discoloration of the XTE specimens compared to CX specimens (p<0.0001), except for the specimens polymerized in the presence of oxygen (group D) and evaluated in WB (p=ns). In Table 2, it is possible to note how the storage in water induced only a minor discoloration effect on both composites when compared to the results attained in the specimens stored in the two staining solutions (red wine and coffee) (ΔE) (p<0.0001). However, after water storage, the specimens in Group D showed a significant increase in ΔE variation compared to Groups A, B, and C (p <0.001). The discoloration results showed that the specimens polymerized in air (group D) were almost always more susceptible to the chromatic changes induced by wine and coffee compared to the other groups.

## 4. Discussion

This study demonstrated that light-curing procedures performed with or without the use of oxygen barriers may influence monomers elution, microhardness, and the color stability of modern universal resin composites.

The HPLC results obtained in this study showed that CX specimens released a significant lower amount of TEGDMA when light-cured using the mylar matrix compared to all the other tested groups. Conversely, the amount of monomers released from XTE was not influenced by the type of light-curing protocol employed to polymerize the specimens, with or without oxygen barriers.

In a previous study performed by Polydorou [21], cylindrical specimens (4.5 mm diameter and 2 mm thickness) created with CX and XTE were light-cured under mylar and, subsequently, stored for 24 h, 7 d, and 28 d in different storage media, including ethanol. They found TEGDMA, DUDMA, and Bis-GMA in XTE eluates, but no elution of monomers for the CX group. The authors attributed such an outcome to the different chemical composition of the two composite materials. Indeed, Ceram X (CX) is a nanohybrid Ormocer-based material, which seems to achieve a great degree of conversion during polymerization reaction, so that low monomer release and high biocompatibility is accomplished [22]. The results of our current study are in disagreement with those latter ones, as we observed that the polymerization of CX was dependent on the type of light-curing strategy employed; both tested resin composites released a micromolar amount of methacrylates.

Removal of the outer layer of resin composites affected by oxygen inhibition polymerization is usually required via finishing procedures; this is to produce a harder, more resistant, and more esthetically acceptable surface [23]. However, several studies have shown that a smoother and harder surface is obtained even when resin composites are light-cured in the absence of oxygen using a mylar matrix [24–26].

The hardness of resin composites can be affected by several elements, such as organic and inorganic composition, filler load, and degree of polymerization [27, 28]. Our results showed that the two resin composites (XTE and CX) used in this study had no significant difference in terms of microhardness when light-cured in the presence of glycerin, argon gas, or air (Figure 6). Nevertheless, CX polymerized using the mylar matrix reached significantly lower microhardness (VHN) than those achieved by CX polymerized in any other condition. The highest VHN was achieved when CX was polymerized in the presence of argon. Likewise, XTE reached the maximum VHN values when cured in argon atmosphere. Accordingly, the first hypothesis of this study must be in part accepted, although hardness and monomers elution seem to be also correlated with the chemical composition of the resin composite selected for clinical restorations.

Composite discoloration may be influenced by many factors, such as degree of conversion during light-curing procedures sorption and solubility and organic and inorganic chemical composition [9]. This current study showed that the ΔE values of CX were often lower than those obtained with XTE; again, this may be correlated with the fact that CX is a nanohybrid composite, while XTE is a nanofilled composite. A study of Ergücü [29] compared the color stabilities of five different composites and observed that when these were exposed to coffee for one week, CX and XTE light-cured using a mylar matrix showed a significant increase in discoloration compared to the other tested composite materials. Moreover, Celik [30] showed that CX was affected by greater color changes than XTE when immersed in three different mouth-rinse solutions.

However, in all cases, the specimens of this study light-cured in the presence of oxygen with the use of no oxygen guard had the highest level of discoloration in all solutions. Conversely, light-curing procedures performed using mylar provided the best results in terms of discoloration. Thus, the second hypothesis must be accepted as the discoloration seems to be correlated with the type of light-curing procedures used to polymerize the modern resin composites.

It is important to consider that the experimental design used in this study for the discoloration assessment of the composites after 28 d of incubation in different solutions may correspond to aprox. 2.5 yr of aging* in vivo* (24 h in vitro staining corresponds to 30 d* in vivo*) [31], considering only the time as variable. More recent studies have shown the important role of bacteria and esterase commonly found in the oral environment in the degradation of resin composites. The ester-linkages in Bis-GMA and TEGDMA composites are subjected to hydrolysis when exposed to enzymes and an esterase, produced by* Streptococcus mutans*, seems to be partly responsible for this intraoral degradation [32]. After exposure to esterase enzyme, a nanofilled composite, Filtek supreme plus, showed 57% reduction in the tensile diametral strength and 46% in elasticity [33].

Moreover, the evaluation of ΔE values both in white background (WB) and in black background (BB) can simulate two different clinical conditions; BB can be associated with a class IV restoration in which there is a dark background behind the composite, while WB is supposed to reproduce class-one restorations surrounded by dental tissue [34].

## 5. Conclusions

Color stability and discoloration of resin composites may be influenced by material type and light-curing strategy. The most appropriate composite should be selected not only for its handling or for its mechanical properties, but also for its color stability, especially if this will be used in esthetic areas. Furthermore, when a clinician is not able for some reasons to properly finish and polish composite restorations, the application of a light-curing protocol performed in the absence of oxygen may improve the chemicophysical properties, as well as the polishability of resin composites. The use of glycerin or argon gas may be suitable for light-curing procedures of occlusal surface in posterior teeth, as well as in all those zones of the composite restoration that cannot be covered by a mylar matrix. However, the advantages of performing light-curing procedures in argon atmosphere should be further investigated.

## Figures and Tables

**Table 1:** Study materials and their composition.

	Shade	Matrix	Filler	Composite type	Wt. %	Manufacturer
CeramX Universal (CX)	A2	Bis-EMA; TEGDMA	SphereTEC™ (ø 3,50 ÷15 *μ*m) non-agglomerated barium glass (ø 3,50 ÷ 0.6 *μ*m) and ytterbium fluoride (ø 3,50 ÷ 0.6 *μ*m).	Nano-hybrid-composite with pre-polymerized fillers	77-79	Dentsply

Filtek Supreme XTE	A2	Bis-GMA TEG-DMA UDMA Bisphenol A-PEGDMA	silica nanofiller(ø= 5-75 nm), zirconia/silica nanocluster (ø=0.6-1.4 *μ*m),	nanofilled composite	72,5	3M ESPE

**Table 2:** Mean ± standard deviation (SD) of the color change (ΔE) of the materials. Water (dH_2_O), Red Wine (RW), Coffee (CF). Horizontal, different superscript letters indicate significant difference in ΔE between same storage solutions for the different polymerization conditions. Exceptions to this statement are clearly shown in the table (ns: not significant). Vertical, different capital letters indicate significant difference in ΔE between different materials in same storage conditions.

BB	dH_2_O	RW	CF	dH_2_O	RW	CF	dH_2_O	RW	CF	dH_2_O	RW	CF

CX	Group A			Group B			Group C			Group D		

Mean ± SD	2.45± 0.78	19,17± 0.47^a^ A vs B ns *E*	13,85± 0.86^a^ *E*	1,34± 0.54	18.70± 3.06^c^ E	18.1± 1.6^c^ *E*	1.56± 0.35	43.93± 4.10^e^ E	22.01± 1.74^e^ E	4.07± 1.81	36.99± 1.04^b^ E	28.25± 0.97^b^ E

XTE	Group A			Group B			Group C			Group D		

Mean ± SD	2,20± 1.1	36.64± 2.73^a^ **F**	26.67± 2.11^a^ F	0.93± 0.3	44.35± 6.97^c^ F	37.2± 4.65^c^ B vs C ns **F**	1.86± 0.64	50.89± 0.87^e^ F	37.51± 2.61^e^ **F**	6.29± 2.08	65.35 ±0.84^b^ F	54.88± 6.7^b^ **F**

WB	dH_2_O	RW	CF	dH_2_O	RW	CF	dH_2_O	RW	CF	dH_2_O	RW	CF

CX	Group A			Group B			Group C			Group D		

Mean ± SD	2.11± 0.24	20.41± 0.11^a^ *A vs B ns* *G*	15.27± 1.36^a^ *A vs B* *ns* *G*	0.86± 0.15	18.25± 1.17^c^ *G*	16.5± 0.85^c^ G	1.58± 0.17	36.86± 0.99 *G*	23.38± 0.59^e^ G	4.24± 0.55	38.64± 0.12^b^ *D vs C ns*	28.89± 0.37^b^

XTE	Group A			Group B			Group C			Group D		

Mean ± SD	1.09± 0.17	35.00± 0.98^a^ H	27.80± 0.67^a^ H	1.15± 0.06	45.80± 2.33 H	38.5± 2.12^c^ *B vs C ns* *H*	1,66± 0.16	52.54± 0.65^e^ C vs D ns H	39.02± 1.04^e^ *H*	7.94± 0.87	48.86± 5.49^b^	58.07± 2.64^b^

## Data Availability

The data used to support the findings of this study are available from the corresponding author upon request.
